# PRDX6-iPLA2 aggravates neuroinflammation after ischemic stroke via regulating astrocytes-induced M1 microglia

**DOI:** 10.1186/s12964-024-01476-2

**Published:** 2024-01-29

**Authors:** Li Peng, Yanyan Ji, Yixin Li, Yan You, Yang Zhou

**Affiliations:** 1 Department of Pathology, College of Basic Medicine, Chongqing Medical University, Chongqing, People’s Republic of China; 2 Molecular Medicine Diagnostic and Testing Center, Chongqing Medical University, Chongqing, People’s Republic of China; 3 Department of Pathology, the First Affiliated Hospital of Chongqing Medical University, Chongqing, People’s Republic of China; 4 The Center for Clinical Molecular Medical Detection, The First Affiliated Hospital of Chongqing Medical University, Chongqing, People’s Republic of China; 5 Department of Pathology, The Second Affiliated Hospital of Chongqing Medical University, Chongqing, People’s Republic of China

**Keywords:** PRDX6-iPLA2, Astrocytes, Microglia, Nox2, Drp1‑ dependent mitchondrial fission

## Abstract

The crosstalk between astrocytes and microglia plays a pivotal role in neuroinflammation following ischemic stroke, and phenotypic distribution of these cells can change with the progression of ischemic stroke. Peroxiredoxin (PRDX) 6 phospholipase A2 (iPLA2) activity is involved in the generation of reactive oxygen species(ROS), with ROS driving the activation of microglia and astrocytes; however, its exact function remains unexplored. MJ33, PRDX6^D140A^ mutation was used to block PRDX6-iPLA2 activity in vitro and vivo after ischemic stroke. PRDX6^T177A^ mutation was used to block the phosphorylation of PRDX6 in CTX-TNA2 cell lines. NAC, GSK2795039, Mdivi-1, U0126, and SB202190 were used to block the activity of ROS, NOX2, mitochondrial fission, ERK, and P38, respectively, in CTX-TNA2 cells. In ischemic stroke, PRDX6 is mainly expressed in astrocytes and PRDX6-iPLA2 is involved in the activation of astrocytes and microglia. In co-culture system, Asp140 mutation in PRDX6 of CTX-TNA2 inhibited the polarization of microglia, reduced the production of ROS, suppressed NOX2 activation, and inhibited the Drp1-dependent mitochondrial fission following OGD/R. These effects were further strengthened by the inhibition of ROS production. In subsequent experiments, U0126 and SB202190 inhibited the phosphorylation of PRDX6 at Thr177 and reduced PRDX6-iPLA2 activity. These results suggest that PRDX6-iPLA2 plays an important role in the astrocyte-induced generation of ROS and activation of microglia, which are regulated by the activation of Nox2 and Drp1-dependent mitochondrial fission pathways. Additionally, PRDX6-iPLA2 activity is regulated by MAPKs via the phosphorylation of PRDX6 at Thr177 in astrocytes.

## Introduction

Following ischemic stroke, reperfusion and the release of damage-associated molecular patterns (DAMPs) lead to a series of pathological changes, including oxidative stress, excitotoxicity, neuroinflammation, and apoptosis [5]. Oxidative stress always occurs when there is an imbalance between the oxidant and antioxidant levels in the cell, resulting in an increase in reactive oxygen species (ROS) [42]. Interestingly, excess ROS interact with neuroinflammation and collectively regulate secondary brain injury after stroke [10].

As the most abundant cell type in the central nervous system (CNS), astrocytes are one of the most prominent contributors to balancing oxidative stress and regulating inflammatory responses in the central nervous system following ischemic stroke [36]. Contrastingly, astrocytes are rich in GSH and GSH metabolism-related enzymes that balance oxidative stress [21]. However, activation of astrocytes could also release large amounts of free radicals through various pathways to promote the activation of inflammatory pathways [1, 27, 42]. Although the specific role of astrocytes remains controversial, there is no doubt that their role in ischemic stroke is important and complex. In some CNS diseases, reactive microglia can be transformed into the M1 phenotype by activated astrocytes [25]. And M1 proinflammatory microglia can further contribute to severe inflammatory responses and aggravate brain injury [18]. All these results indicate that astrocytes could not only promote neuroinflammation in ischemic stroke by themselves, but also enhance its pro-inflammatory effect by interfering with microglia. Therefore, the regulation of inflammatory signaling by astrocytes may eventually determine the outcome of CNS inflammation to a large extent. However, the pathological mechanisms underlying this regulation are not completely understood.

Peroxiredoxin 6 (PRDX6) is a “moonlight protein” with glutathione peroxidase and calcium-independent phospholipase A2 (iPLA2) activity [12]. Although PRDX6 could act as an antioxidant by limiting oxidative stress via peroxidase activity. However, it also could increase large amount of reactive oxygen species (ROS) via iPLA2 activity [7]. Interestingly, the dual function of PRDX6 appears to be consistent with its controversial roles in astrocytes. What is more, accumulating evidence has demonstrated that PRDX6 is mainly expressed in astrocytes [29]. Therefore, this interesting phenomenon led us to speculate that PRDX6, with its specific function and structure, may be a key molecule involved in the controversial role of astrocytes in stroke. Chatterjee et al. found that inhibiting PRDX6-iPLA2 activity suppressed the activation of NADPH oxidase (NOX2), which is primarily responsible for the generation of ROS associated with mitochondrial fission and inflammation [7, 49]. Notably, ROS can also induce microglial activation and perturb the kinetics of the M1/M2 shift [47]. These results indicate that the role of PRDX6 in regulating glial cells is complex and unclear, requiring further exploration. This study aimed to explore whether PRDX6-iPLA2 regulates the function of astrocytes and astrocyte-induced microglial polarization in response to ischemic stroke and to identify the potential underlying mechanisms.

## Materials and methods

### Experimental animals and reagents

Adult male Sprague-Dawley rats (weighing 230–250 g) were used for the in vivo study and obtained from the Animal Experimental Center of Chongqing Medical University. The animals were cared for in strict accordance with the Guide for the Care and Use of Laboratory Animals (NIH Publication No. 85 − 23, revised 1996). Each rat was housed in a single cage with free access to food and water, and randomly divided into different groups. Room temperature was maintained at 22 ± 2 °C.

All reagents and materials were obtained as detailed in the supplementary materials.

### Experimental design

**Fig. 1:**
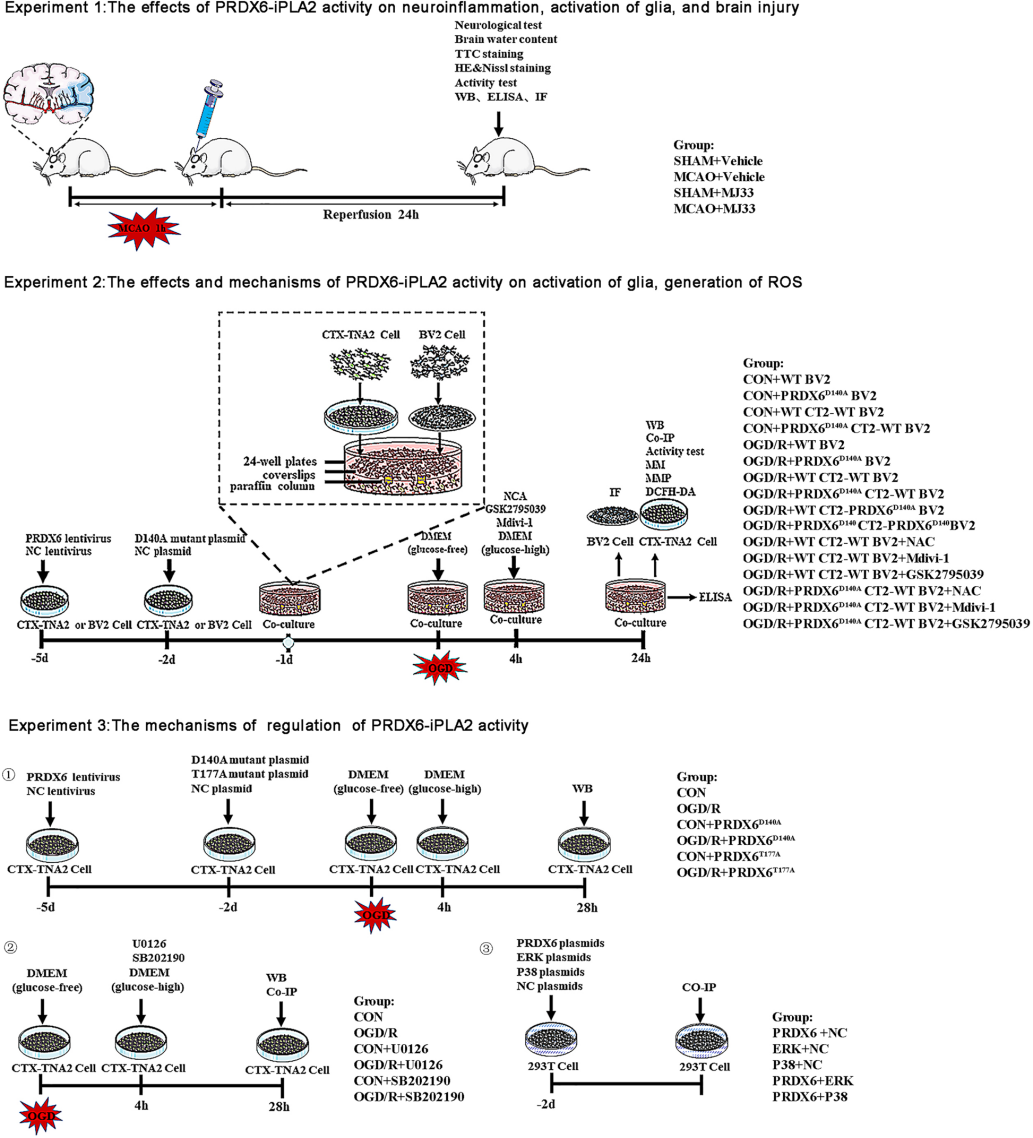
Schematics of ischemic stroke model and experimental design

A schematic of the experimental design, including the number of animals, groups, and experimental methods, is shown in Fig. 1.

### Middle cerebral artery occlusion/Reperfusion (MCAO/R) model and MJ33 treatment

Transient MCAO/R was induced as previously described by our laboratory [31]. Briefly, adult male SD rats were deprived of food and water for 8 h before operation, anesthetized with sodium pentobarbital (50 mg/kg, intraperitoneal injection), and then placed on a heating pad to maintain their body temperature at 37 ± 0.5 °C for the operation. A nylon filament was inserted into the left middle cerebral artery. The filament was removed after 1 h of ischemia to allow for reperfusion. Intraoperative local cerebral blood flow (CBF) was monitored using a laser Doppler flowmeter (Periflux System 5000; Perimed, Sweden). After 24 h of reperfusion, neurological deficit score assays were used to evaluate the success of the MCAO model. Brain tissues were subjected to western blot analysis, immunoprecipitation, and immunohistochemistry. The sham-operated animals underwent the same surgery as the MCAO/R group, except they were not subjected to MCAO.

We dissolved MJ33 in 10% DMSO and 90% saline. We injected 0.5 µmol/kg MJ33 into MCAO model rats via the tail vein after they awoke post-surgery. An identical volume of 10% DMSO/90% saline was injected into the tail vein of control rats.

### Evaluation of neurological deficits

We measured neurological deficits in rats 24 h after MCAO/R using the Longa method as previously described [31]. The scoring system was as follows: grade 0, no neurological deficits; grade 1, unable to extend the contralateral forelimb entirely; grade 2, circling to the right; grade 3, falling to the contralateral side; grade 4, no spontaneous autonomic activity or loss of consciousness; and grade 5, death.

### Quantification of infarct volume

After MCAO/R, the rats were anesthetized with an overdose of pentobarbital and their brains were rapidly removed. Brains were sliced into five 2-mm-thick sections after freezing at − 20 °C for 20 min. The sections were stained and photographed, and the volume was analyzed as previously described [31].

### Measurement of brain edema

We measured brain water content in rats after MCAO/R. The rats were euthanized 24 h after reperfusion, and their brains were rapidly removed and divided into ipsilateral and contralateral hemispheres. Both hemispheres were weighed to obtain the wet weight, then dried at 100 °C for 48 h to obtain the dry weight. Brain water content was calculated as follows: (wet weight − dried weight) / wet weight × 100%.

### Hematoxylin‒eosin (H&E) and Nissl staining

Rat brain tissues were dehydrated and paraffin-embedded after fixation in paraformaldehyde for 24 h. Then, 5-µm-thick coronal sections were stained with hematoxylin‒eosin (HE) or 0.1% cresyl violet (Nissl stain) for H&E or Nissl staining, respectively, based on the manufacturer’s guidance. Pathological changes were assessed by microscopy.

### Electron microscopy

After MCAO/R, the rats were anesthetized with an overdose of 1% pentobarbital, and their brains were quickly removed after transcardial perfusion with a solution of 4% paraformaldehyde and 2.5% glutaraldehyde in PBS. Cortical tissue samples were sliced into 1-mm^3^ pieces. The mitochondrial ultrastructure was analyzed using transmission electron microscopy (JEM-1400PLUS, Tokyo, Japan).

### Immunocytochemistry and immunohistochemistry

Frozen sections (8 μm thick) and cells on coverslips were fixed in 4% paraformaldehyde and washed with PBS. After blocking in 5% bovine serum albumin (BSA) at 37 °C for 1 h, frozen sections and cells were incubated with the following primary antibodies overnight at 4 °C. Next day, the samples were then incubated after three cycles of PBS washing with the corresponding fluorescence-conjugated secondary antibodies for 1 h at 37 °C. Three PBS saline washes were performed. Micrographs were analyzed using the ImageJ software.

### Cell culture and oxygen and glucose deprivation/Reoxygenation (OGD/R) treatment

The immortalized rat astrocyte cell line CTX-TNA2 was purchased from Shanghai Saibai Kang Biotechnology Company (cat. #iCell-r008, Shanghai, China) and immortalized mouse microglial cell line BV2 was purchased from Shanghai Qingqi Biotechnology (cat. # BFN60810727, Shanghai, China). The CTX-TNA2 were plated directly on the plastic surface in 24-well plates. BV2 were plated onto poly-L-lysine-coated, PBS pre-equilibrated, 14-mm glass coverslips in 24-well plates.

Non-contact BV2-CTX-TNA2 co-cultures were established as shown in Fig. 1. Briefly, when BV2 cells were almost adhered to a poly-L-lysine-coated coverslips on paraffin column without CTX-TNA2 cells, they were incubated with CTX-TNA2 cells monolayers in a manner that did not allow physical contact between the two cell types. The paraffin column was not injurious to cultures. CTX-TNA2 and BV2 cells were cultured in DMEM supplemented with 10% fetal bovine serum, and 1% penicillin-streptomycin in a humidified 37 °C incubator with 5%CO_2_ respectively. To establish an in vitro MCAO/R model, OGD/R was induced as previously described [31]. Briefly, CTX-TNA2 and BV2 cells were established in glucose-free DMEM and were transferred to an incubator with 94% N_2_, 1% O_2_, and 5% CO_2_ at 37 °C for 4 h. Next, glucose-free DMEM was replaced by DMEM supplemented with 10% fetal bovine serum, and 1% penicillin-streptomycin and the cells were grown in an incubator with 5% CO_2_ at 37 °C and harvested after 24 h for further analysis.

HEK293T cells (Human embryonic kidney cell line, Thermo Fisher) were maintained in a humid incubator in DMEM supplemented with 10% FBS, 1% GlutaMAX, and 1x Penicillin-Streptomycin. Cells were enzymatically passaged at 90% confluence by trypsinization. Incubator conditions: 5% CO_2_, 37℃.

### Drug treatments

For CTX-TNA2 cell cultures, Mdiv1-1, GSK2795039, NAC, SB202190, and U0126 were dissolved into DMSO and then added in DMEM culture medium at various concentrations (Mdivi-1:50 µM, GSK2795039:25 µM, NAC:10 µM, SB202190:30µM, U0126:10 µM) after 4 h of OGD.

### Construct PRDX6-iPLA2 ^Asp140^ mutations

The lentivirus was supplied by Neuron Biotech (Shanghai, China). We knocked down PRDX6 using a lentivirus (NCBI accession no. NM_053576.2 → NP-446028.1; shRNA, 5′-ACAGCCCGTGTGGTATTCAT-3′). Three days after lentiviral transfection, plasmid constructs expressing PRDX6 without iPLA2 activity due to the Asp140 gene mutation were transfected into CTX-TNA2 cells. iPLA2 and glutathione peroxidase activity assay kits were used to detect iPLA2 and GPx activities of PRDX6.

### Plasmid treatments in HEK293T cells

Plasmids containing PRDX6, ERK, P38, or the negative control together with Lipofectamine 2000 (Invitrogen, Carlsbad, CA, USA) were added to the media of HEK293T cells according to the manufacturer’s instructions. After 48-h of incubation, the co-immunoprecipitation experiment were performed.

### Molecular docking of protein and protein

#### Preparation of protein structure

MAPK-38P, ERK and PRDX6 target protein structures for homology modeling were obtained using the HDOCK online server (http://hdock.phys.hust.edu.cn/; template:1LB6-95.0%, 4NEU-75.9%). The MAPK-38P (PDB ID:3MVL), ERK (PDB ID: 1GOL) and PRDX6 (PDB ID:1PRX) protein crystal structure were obtained from the RCSB database (https://www.rcsb.org/). All protein structures were processed on the molecular operating environment platform (MOE 2019.1), and the position was Amber10, including the removal of water and ions, protonation, addition of missing atoms and complementation of missing groups, and minimization of protein energy.

#### Molecular docking

The HDOCK software was used to set the protein as rigid, the docking contact site as the full surface, and the generated conformation after docking was set at 100. The most negative energy conformation was selected using a scoring function and visualized using Pymol 2.1 software.

### Detection of ROS production

Intracellular ROS production in CTX-TNA2 cells was detected using the fluorescent probe 2′,7′-dichlorofluorescein diacetate (DCFH-DA). CTX-TNA2 cells with the indicated treatments were incubated with10 µM DCFHDA in the dark for 30 min at 37 °C. After washing three times with PBS, the cells were scanned using a microplate reader.

### Co-immunoprecipitation assays

Cell lysates were incubated with precoupled antibodies bound to protein G beads overnight at 4 °C. The beads were centrifuged and washed four times with lysis buffer. The complex was resuspended in PBS and used to detect endogenous interactions between the target antibody and other proteins by western blotting.

### Western blot analysis

CTX-TNA2 cells and ischemic brain tissue were homogenized in RIPA buffer containing (1 mM) fluoride. Equal amounts of protein lysates (per lane 50 µg) were separated by SDS-PAGE and electrotransferred onto PVDF membranes. The membranes were blocked with 5% nonfat milk for 2 h at RT and incubated with primary antibodies at 4 °C overnight. The next day, membranes were incubated with horseradish peroxidase-conjugated secondary antibodies for 2 h at RT. An imaging densitometer (Bio-Rad) was used to evaluate the band densities, and ImageJ software was used to quantify the gray values of the bands.

### Mitochondrial morphology analysis

After OGD/R, CTX-TNA2 cells were incubated with prewarmed staining solution containing the MitoTracker® probe for 30 min at 37 °C. Then cells were washed with pre-warmed growth medium, and fixed with pre-warmed growth medium containing 4% paraformaldehyde for 15 min at 37 °C. The cells were permeabilized in PBS containing 0.2% Triton X-100 for 10 min. A laser scanning confocal microscope was used to categorize mitochondria based on length: elongated (> 3 μm), tubular (1–3 μm), and fragmented (< 1 μm).

### Mitochondrial membrane potential

The mitochondrial transmembrane potential was assessed using a JC-1 kit. CTX-TNA2 cells were treated according to the manufacturer’s instructions. Images were captured using a microscope and analyzed using ImageJ software.

### ELISA Analysis

Total protein from ischemic brain tissue or cultured cells was prepared using RIPA buffer containing PMSF. We used ELISA kits to measure the levels of Arg1, iNOS, CD86, IL-10, IL-6, IL-1α, TNF-α, and IL-1β in cells. The levels of these factors in cell lysates were assessed according to the manufacturer’s instructions. The absorbance was measured at 450 nm.

### Statistical analysis

All data are shown as the mean ± standard error of the mean, and statistical analysis was conducted using GraphPad Prism software (version 6.0; La Jolla, CA, USA). Statistical comparisons were performed using one-way analysis of variance (ANOVA) followed by Tukey’s test. Statistical significance was set at *p* < 0.05.

## Results

### Inhibition of PRDX6-iPLA2 reduces ischemic brain injury

**Fig. 2:**
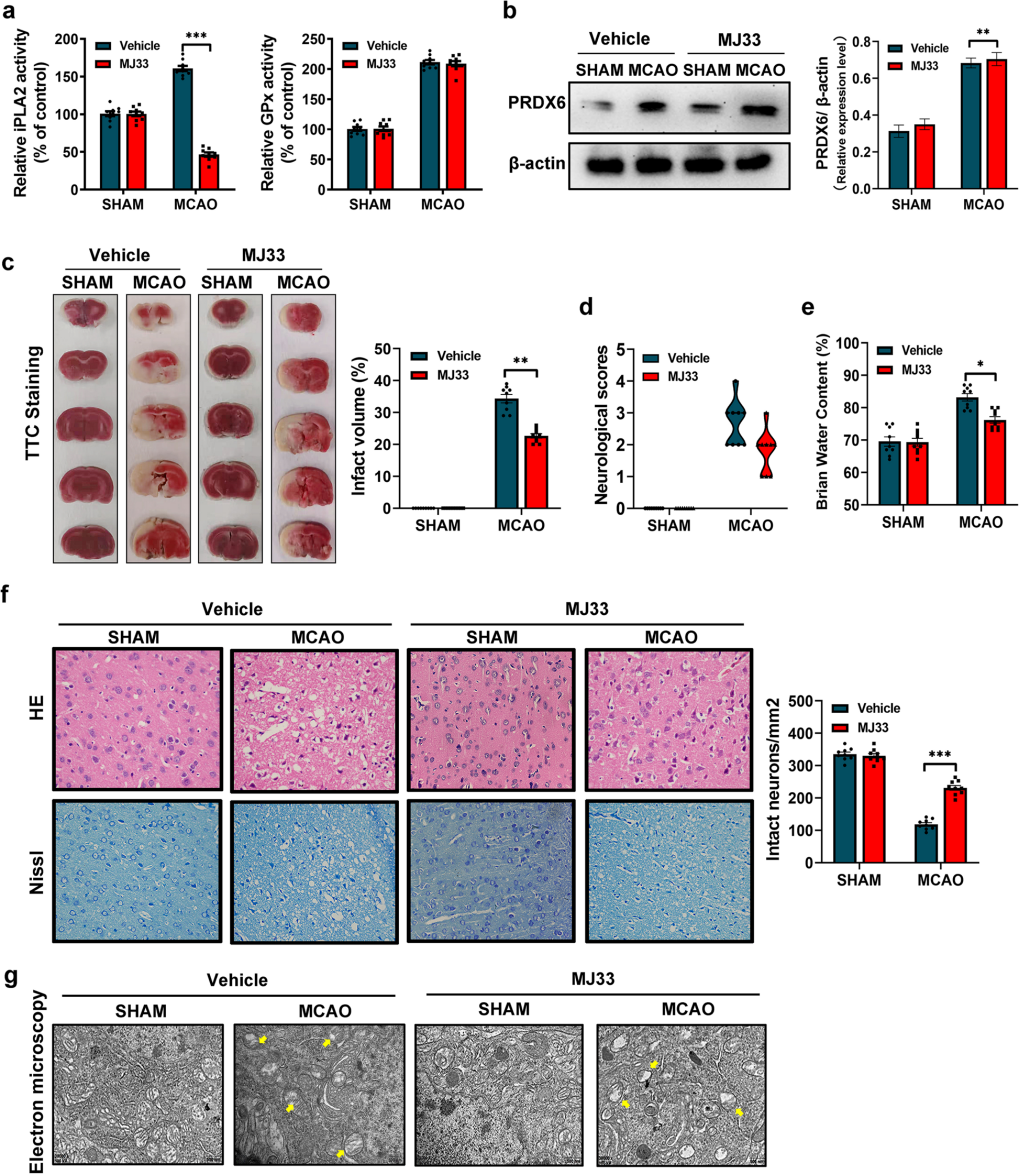
Inhibition of PRDX6-iPLA2 reduces ischemic brain injury. **a** iPLA2 activity and GPx activity in rats. **b** Western blot analysis of PRDX6. **c** TTC staining and Infarct volume of the brain. **d** Neurological deficit scores. **e** Brain water content. **f** HE and Nissl staining (× 400) and histograms showing the number of intact neurons. **g** Mitochondrial ultrastructure. Values are mean ± SEM, * *p*  < 0.05, ** *p*  < 0.01, *** *p*  < 0.001. *n*  = 6 per group

To explore the potential role of PRDX6-iPLA2 in stroke, MJ33, an inhibitor of the iPLA2 activity of PRDX6, was used to inhibit PRDX6-iPLA2 after 60 min of MCAO, a rat model for stroke, then followed by 24 h of reperfusion. First, we verified that iPLA2 and GPx activity remarkably increased in rats after cerebral I/R injury. MJ33 remarkably suppressed iPLA2 activity but not GPx activity, with no effect on the expression of PRDX6 in rats after MCAO/R (Fig. 2a, b). The extent of cerebral infarction was measured 24 h after the onset of MCAO/R. Compared to control rats, the stroke volume was significantly reduced after MJ33 treatment (Fig. 2c). Reduced neurological damage and brain water content were observed in the MJ33 group compared to the MCAO group (Fig. 2d, e). Compared with the sham group, HE staining revealed disorderly cytoplasm, edema, and karyopyknosis of neurocytes in the MCAO group, and Nissl staining showed decreased Nissl bodies in the MCAO group. These changes were significantly reversed by MJ33 treatment (Fig. 2f). Morphological changes in the mitochondria were evident in response to different types of injury. Therefore, a projection electron microscope was used to assess the mitochondrial ultrastructure (Fig. 2g). Compared to the sham group, the mitochondrial outer compartment was substantially wider in the MCAO group. However, MJ33 treatment reduced mitochondrial swelling compared with that in the MCAO group. These results indicate that ischemic stroke promotes the activation of PRDX6-iPLA2, causing brain injury. Inhibition of PRDX6-iPLA2 activity reduces ischemic brain injury.

###  Inhibition of PRDX6-iPLA2 influences astrocyte and microglia activation in the ischemic penumbra

**Fig. 3:**
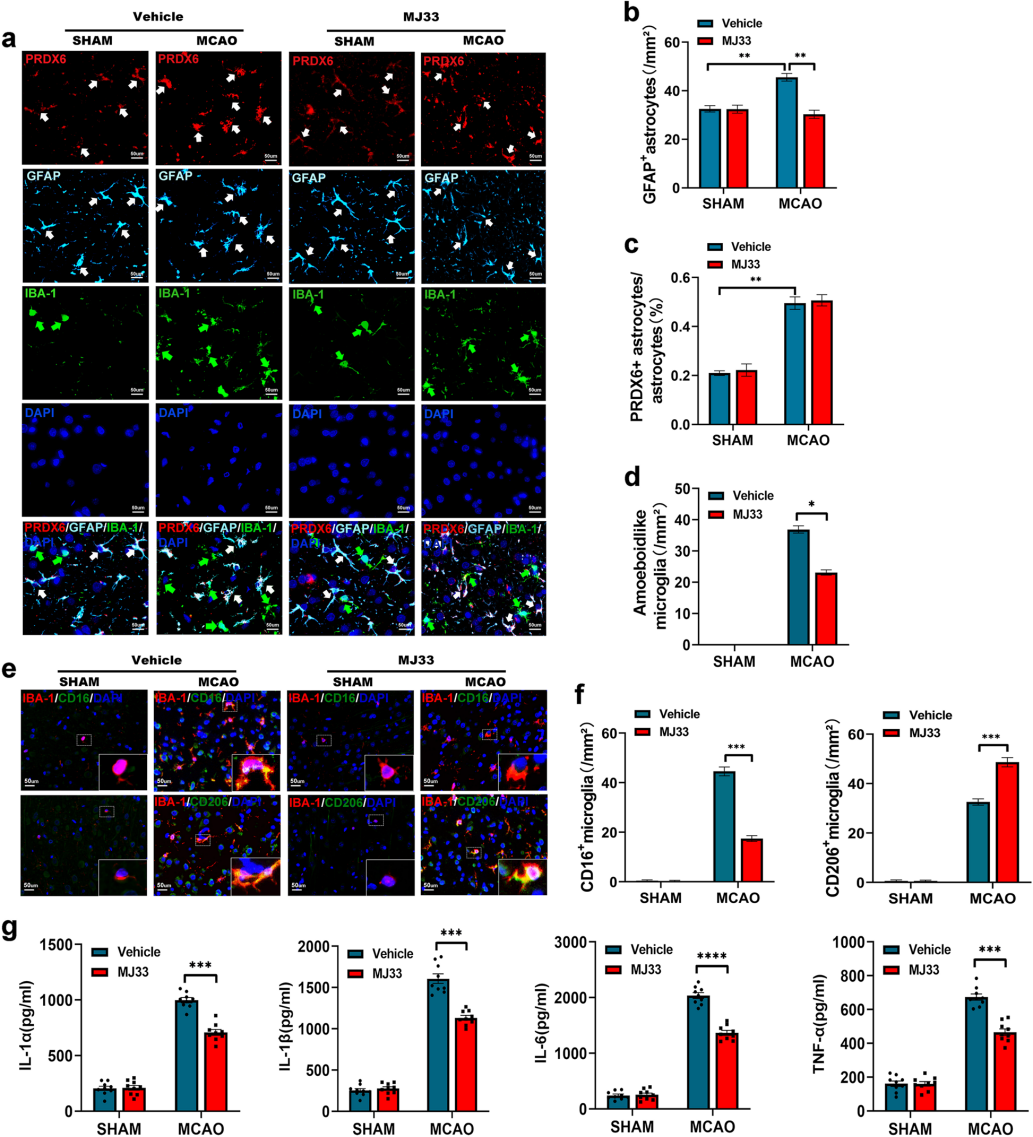
Inhibition of PRDX6-iPLA2 influences astrocyte and microglia activation in the ischemic penumbra. **a** Immunocytochemistry of Iba-1 staining for microglia. Immunocytochemistry of GFAP staining for astrocytes. PRDX6 expression and location was measured by immunohistochemistry in rats after treatment with MJ33. Cell nuclei were stained with DAPI (× 400). **b** Histogram reveals the number of the activated GFAP + astrocytes. **c** Histogram shows PRDX6 + astrocytes. **d** Histogram reveals the amoebodilike microglia. **e** Immunocytochemistry of Iba-1 staining for microglia. CD16 and CD206 expression were detected by immunohistochemistry in microglia after treatment with MJ33. Cell nuclei were stained with DAPI (× 400). **f** Histogram reveals the number of CD16 + microglia and CD206 + microglia. **g** Quantification of IL-1α, IL-1β, IL-6, and TNF-α in rats by ELISA. Values are mean ± SEM, * *p*  < 0.05, ** *p*  < 0.01, *** *p*  < 0.001, **** *p*  < 0.0001. *n*  = 6 per group

Since activated glia plays an important role in the progression of post-ischemic neuroinflammation, affecting the outcome of cerebral ischemic injury [37], we characterized the impact of PRDX6-iPLA2 inhibition on major neuroinflammation-associated glial cells (astrocytes and microglia/infiltrated macrophages) after MCAO/R using immunostaining. First, we used multicolor immunostaining and LSCM imaging to analyze the cellular localization of the PRDX6 protein in neuroinflammation-associated glial cells. We found that activated GFAP^+^ astrocytes were localized to the ischemic penumbra after MCAO/R. Meanwhile, PRDX6 mostly colocalized with GFAP^+^ astrocytes, and its expression is increased in GFAP^+^ astrocytes in the ischemic penumbra. The number of activated GFAP^+^ astrocytes was suppressed in the ischemic penumbra after inhibition of PRDX6-iPLA2. However, MJ33 treatment had no effect on the expression of PRDX6 in the astrocytes of the ischemic penumbra (Fig. 3a, b).

**Fig. 4:**
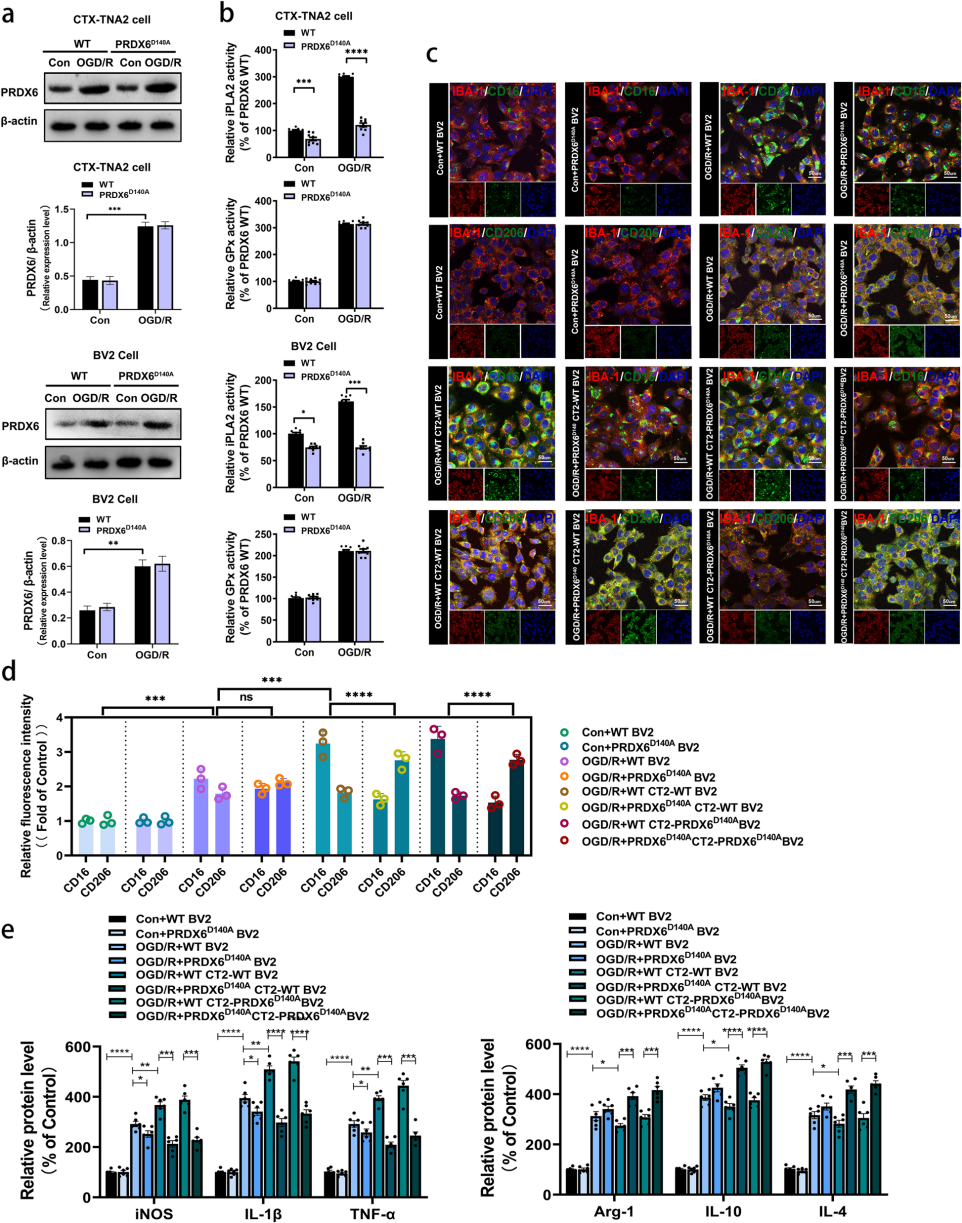
Inhibition of astrocytic PRDX6-iPLA2 influences microglia/infiltrated macrophages polarization. **a** Western blot analysis of PRDX6 in CTX-TNA2 cells or BV2 cells. **b** iPLA2 activity in CTX-TNA2 cells or BV2 cells. GPx activity in CTX-TNA2 cells or BV2 cells. **c** Immunocytochemistry of Iba-1 staining for microglia. CD16 and CD206 expression were detected by immunohistochemistry in BV2 cells. Cell nuclei were stained with DAPI (× 400). **d** Histogram reveals fluorescence intensity of CD16 and CD206. **e** Quantification of iNOS, IL-1β, TNF-α, Arg-1, IL10 and IL-4 in BV2 cells by ELISA. Values are mean ± SEM, * *p*  < 0.05, ** *p*  < 0.01, *** *p*  < 0.001, **** *p*  < 0.0001

Similarly, we found that the activated amoeboid-like microglia/infiltrated macrophages were localized in the ischemic penumbra and were significantly increased after ischemic stroke. Treatment with MJ33 reduced the number of amoeboid-like microglia/infiltrating macrophages after ischemic stroke. Interestingly, we did not detect visible PRDX6 in IBA1 + microglia/infiltrating macrophages in the ischemic penumbra. Immunostaining for PRDX6 and IBA1 did not show colocalization in any of these groups (Fig. 3a, c). However, some studies have shown the expression of PRDX6 has also been detected by western blot in the microglia in vitro [9, 35]. Astrocyte cell line CTX-TNA2 cell and microglial cell line BV2 was used to analyze the expression of PRDX6 after OGD/R, respectively. CTX-TNA2 cells and BV2 cells showed a similar increase after OGD/R. However, the content of PRDX6 in astrocytes is visible higher than that in BV2 cells under resting state, and the upregulation of PRDX6 in astrocytes is 2-fold that of microglia after OGD/R (Fig. 4a). This may be why PRDX6 and IBA1 did not show colocalization in microglia/infiltrated macrophages in immunostaining. Here, we demonstrated that PRDX6 is mainly expressed in astrocytes, but less expression in microglia. Even though, the inhibition of PRDX6-iPLA2 activity could both affect the activation of astrocytes and microglia in the ischemic penumbra.

Next, we used ELISA to detect the levels of pro-inflammatory cytokines and immunostaining to measure the number of pro- and anti-inflammatory microglia [50]. Pro-inflammatory cytokines, including IL1α, IL1β, IL6, TNFα (Fig. 3g), and the number of CD16 + and CD206 + microglia (especial CD16 + microglia) (Fig. 3e, f), increased after ischemic stroke. PRDX6-iPLA2 inhibition reduced the levels of these pro-inflammatory cytokines and the number of CD16 + microglia, and increasing the number of CD206 + microglia after ischemic stroke. These results illustrate that PRDX6-iPLA2 participates in the regulation of the inflammatory response and microglia/infiltrated macrophages polarization after ischemic stroke.

### Inhibition of astrocytic PRDX6-iPLA2 influences microglia/infiltrated macrophages polarization

A lentivirus was used to knockdown PRDX6 in CTX-TNA2 cells and BV2 cells, respectively, and the D140A mutant plasmid was transfected to construct the PRDX6^D140A^ CTX-TNA2 cell line and the PRDX6^D140A^ BV2 cell line for subsequent experiments. As shown in Fig. 4b, iPLA2 and GPx activity remarkably increased was markedly upregulated in wild-type BV2 cells and CTX-TNA2 cells following OGD/R. Interestingly, in CTX-TNA2 cells, compared to the control group, the content of PRDX6-iPLA2 activity increased by two times after OGD/R. After the Asp140 mutation in PRDX6, iPLA2 activity decreased about 1.7 times compared to the OGD/R group. In BV2 cells, compared to the control group, the content of PRDX6-iPLA2 activity increased by 0.6 times after OGD/R. After the Asp140 mutation in PRDX6, iPLA2 activity decreased about 1 times compared to the OGD/R group. However, Asp140 mutation in PRDX6 of BV2 cells or CTX-TNA2 cells also had no effect on expression of PRDX6 and GPx activity following OGD/R (Fig. 4a, b). Considering that compared to the control group of the same type of cells, after mutation Asp140 in PRDX6, the reducing content of PRDX6-iPLA2 activity in CTX-TNA2 cells is 1.7 times that of the OGD/R group, while the reducing content of PRDX6-iPLA2 activity in BV2 cells is 1 times that of the OGD/R group, we speculate that the Asp140 mutation in PRDX6 has a greater impact on the PRDX6-iPLA2 activity in astrocytes.

To further explore whether PRDX6-iPLA2 is involved in the polarization of microglia/infiltrated macrophages, and these changes of microglia/infiltrated macrophages is whether related to astrocytes. Monocultured wild-type BV2 cells and PRDX6D^140A^ BV2 cells, and non-contacting co-cultures system of wild-type CT2 -wild-type BV2 cells, PRDX6^D140A^ CT2 -wild-type BV2 cells, wild-type CT2 -PRDX6^D140A^ BV2 cells and PRDX6^D140A^ CT2 -PRDX6^D140A^ BV2 cells were subjected to OGD5h/R24h, then ELISA and multicolor immunostaining was used to analyze the role of astrocytic PRDX6-iPLA2 in the polarization of microglia/infiltrated macrophages. Multicolor immunostaining results showed that the fluorescence intensity of both CD16 and CD206 was remarkably increased in wild-type BV2 cells following OGD/R. Asp140 mutation in PRDX6 of BV2 cells slightly reduced the fluorescence intensity of CD16, mild increased the fluorescence intensity of CD206 after OGD/R (Fig. 4c, d). This conclusion contradicts the results of the reduction of CD16 + microglia in MCAO/R rat due to the inhibition of PRDX6-iPLA2. Thus, we speculate that the regulation of PRDX6-iPLA2 on microglia polarization may be related to the activation of astrocytes. Interestingly, for co-cultures system, compared with WT CT2-WT BV2 group, Asp 140 mutation in PRDX6 of CT2 cells remarkably reduced the fluorescence intensity of CD16 and increased the fluorescence intensity of CD206 in BV2 cells in PRDX6^D^^140A^ CT2 -WT BV2 group after OGD/R. There is no significant difference between wild-type CT2 -wild-type BV2 group and wild-type CT2 -PRDX6^D140A^ BV2 cells group. Similarly, PRDX6^D140A^ CT2 -wild-type BV2 group and PRDX6^D140A^ CT2 -PRDX6^D140A^ BV2 group also have no significant difference. We next measured the expression of pro-inflammatory microglial cell marker iNOS and mediators (IL-1β and TNF-α), and anti-inflammatory microglial cell markers Arg1 and mediators (IL-4 and IL-10) in BV2 cells by ELISA (Fig. 4e). The ELISA results of pro-inflammatory microglial cell marker and mediators were consistent with the fluorescence results of CD16 + microglia, and anti-inflammatory microglial cell markers and mediators were consistent with the fluorescence results of CD206 + microglia. These results illustrate that astrocytic PRDX6-iPLA2 participates in the regulation of the microglia/infiltrated macrophages polarization after ischemic stroke.

### Inhibition of astrocytic PRDX6-iPLA2 influences microglia/infiltrated macrophages polarization through mediating the generation of ROS

**Fig. 5:**
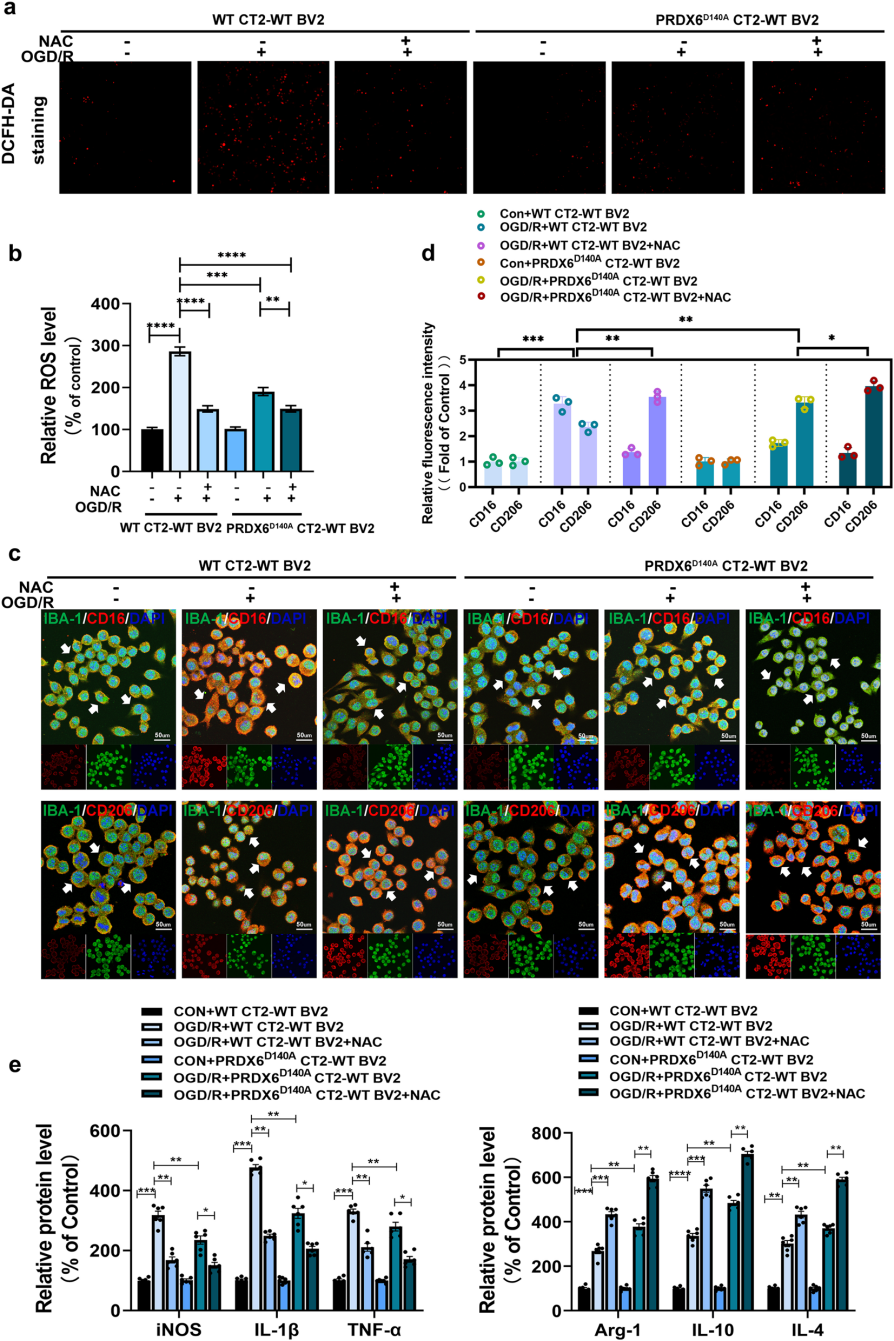
Inhibition of astrocytic PRDX6-iPLA2 influences microglia/infiltrated macrophages polarization through mediating the generation of ROS. **a** DCFH-DA staninig. **b** Quantification of ROS. **c** Immunocytochemistry of Iba-1 staining for microglia. CD16 and CD206 expression were detected by immunohistochemistry in BV2 cells. Cell nuclei were stained with DAPI (× 400). **d** Histogram reveals fluorescence intensity of CD16 and CD206. **e** Quantification of iNOS, IL-1β, TNF-α, Arg-1, IL10 and IL-4 in BV2 cells by ELISA. Values are mean ± SEM, *p < 0.05, **p < 0.01, ***p < 0.001, ****p < 0.0001

To further determine whether PRDX6-iPLA2 in astrocytes is involved in the polarization of microglia/infiltrated macrophages through mediating the generation of ROS, a lentivirus was used to knockdown PRDX6, and the D140A mutant plasmid was transfected to construct the PRDX6^D140A^ CTX-TNA2- WT BV2 co-culture system for subsequent experiments. Compared to the CON + WT CT2-WT BV2 group, multicolor immunostaining results in the OGD/R + WT CT2-WT BV2 group revealed that the fluorescence intensity of CD16 was remarkably increased (Fig. 5c). Compared with the OGD/R + WT CT2-WT BV2 group, the fluorescence intensity of CD16 was reduced in the OGD/R + PRDX6^D140A^ CT2-WT BV2 group. In contrast, the fluorescence intensity of M2 microglia-labeled CD206 showed the opposite results. We next measured the expression of pro-inflammatory microglial cell marker iNOS and mediators (IL-1β and TNF-α), and anti-inflammatory microglial cell markers Arg1 and mediators (IL-4 and IL-10) by ELISA (Fig. 5e). The ELISA results were consistent with the fluorescence results. These results suggest that PRDX6-iPLA2 activity is stimulated by OGD/R in the astrocyte-mediated polarization of microglia/infiltrated macrophages.

Because PRDX6 is vital for the regulation of ROS [7], an ROS activity test was performed to detect the concentration of ROS in CTX-TNA2 cells in CTX-TNA2- WT BV2 co-culture system and their supernatants. ROS generation markedly increased in wild-type CTX-TNA2 cells following OGD/R stimulation. Unlike wild-type cells, the generation of ROS induced by OGD/R was significantly inhibited in PRDX6^D140A^ CTX-TNA2 cells (Fig. 5a, b). Interestingly, previous studies revealed that ROS can activate microglia/infiltrated macrophages and mediate their phenotype [45]. We further explored whether ROS are required for astrocytic PRDX6-iPLA2 mediating the polarization of microglia/infiltrated macrophages after OGD/R. Thus, ROS inhibitor (NAC) was added after OGD. In OGD/R + WT CT2-WT BV2 + NAC group, NAC markedly inhibited ROS generation after OGD/R stimulation, and the ROS content in PRDX6^D140A^ CT2-WT BV2 system after OGD/R was further reduced after treatment with NAC (Fig. 5a, b). These results reveal that the iPLA2 activity of PRDX6 is an upstream mediator of ROS induced by OGD/R in astrocytes.

Next, compared to the OGD/R + WT CT2-WT BV2 group, the multicolor immunostaining results in the OGD/R + WT CT2-WT BV2 + NAC group revealed that the fluorescence intensity of CD16 in the microglia was remarkably decreased. Compared to the OGD/R + PRDX6^D140A^ CT2-WT BV2 group, the fluorescence intensity of CD16 was further reduced in the OGD/R + PRDX6^D140A^ CT2-WT BV2 + NAC group (Fig. 5c, d). Contrastingly, the fluorescence intensity of M2 microglia-labeled CD206 showed the opposite results. The ELISA results were consistent with the fluorescence results (Fig. 5e). These data suggest an essential role of astrocytic PRDX6-iPLA2 activity in regulating ROS generation and mediating the polarization of microglia/infiltrated macrophages induced by ROS.

### Inhibition of astrocytic PRDX6-iPLA2 influences Drp1-dependent mitchondrial fission through mediating the generation of ROS

**Fig. 6:**
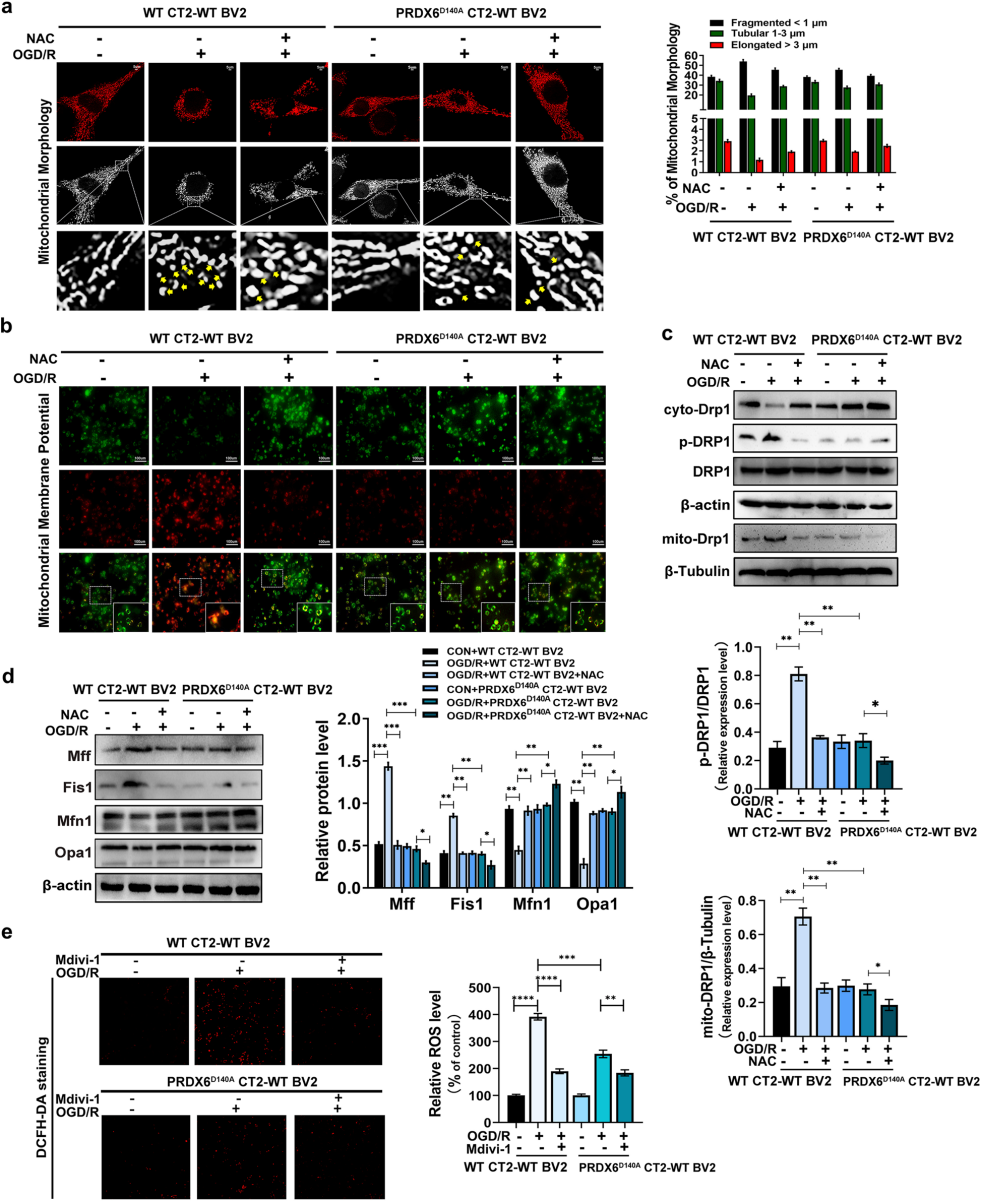
Inhibition of astrocytic PRDX6-iPLA2 influences Drp1-dependent mitochondrial fission through mediating the generation of ROS.** a** Mitochondrial morphology. **b** Mitochondrial membrane potential. **c** Western blot analysis of Drp1, cyto-Drp1, p-Drp1, mitochondrial Drp1 in CTX-TNA2 cells. **d** Western blot analysis of fission-related proteins and fusion-related factors in CTX-TNA2 cells. **e** DCFH-DA staninig and quantification of ROS. Values are mean ± SEM, *p < 0.05, **p < 0.01, ***p < 0.001, ****p < 0.0001

Previous study revealed that ROS are upstream mediators of Drp1-dependent mitochondrial fission or fragmentation in neuropathic pain [51]. To confirm whether PRDX6-iPLA2 in co-culture CTX-TNA2 cells regulates Drp1-dependent mitochondrial fission induced by ROS, mitochondrial morphology and membrane potential were assessed to detect mitochondrial fission or fragmentation in astrocytes after OGD/R (Fig. 6a, b). In OGD/R + WT CT2-WT BV2, the number of fragmented mitochondria significantly increased, and the number of elongated and tubular mitochondria decreased in co-culture CTX-TNA2 cells. NCA treatment reduced the number of OGD/R-induced fragmented mitochondria, whereas the number of elongated and tubular mitochondria increased in co-culture CTX-TNA2 cells. In OGD/R + PRDX6 ^D140A^ CT2-WT BV2, PRDX6^D140A^ CTX-TNA2 cells exhibited a decrease in the number of fragmented mitochondria after OGD/R. Furthermore, treatment with NCA reduced the number of fragmented mitochondria in CTX-TNA2 cells. The mitochondrial membrane potential was significantly decreased in co-culture CTX-TNA2 cells after OGD/R (Fig. 6b). Notably, significant downregulation of mitochondrial membrane potential induced by OGD/R was not observed in PRDX6^D140A^ astrocytes. Treatment with NCA rescued the OGD/R-induced decrease in response to OGD/R.

Mitochondrial fission is mainly regulated by the translocation of DRP1 from the cytoplasm to the mitochondria [41]. We found that OGD/R resulted in the translocation of DRP1 from the cytoplasm to mitochondria in co-culture CTX-TNA2 cells (Fig. 6c). However, the translocation of DRP1 following OGD/R was reversed after treatment with NCA. Interestingly, the changes in Drp1 translocation induced by OGD/R in co-culture PRDX6^D140A^ CTX-TNA2 cells were consistent with those observed in OGD/R + WT CT2-WT BV2 + NAC group. Furthermore, the translocation of DRP1 from the cytoplasm to the mitochondria is mainly mediated by the phosphorylation of Drp1 at Ser616 [17]. Results revealed that OGD/R promoted the phosphorylation of Drp1 at Ser616 in co-culture CTX-TNA2 cells, and this effect was reversed by treatment with NCA or transfection with the PRDX6^D140A^ mutant in co-culture CTX-TNA2 cells (Fig. 6c). Compared to OGD/R + PRDX6^D140A^ CT2-WT BV2 group, the phosphorylation of Drp1 at Ser616 was further reduced after treatment with NCA in co-culture CTX-TNA2 cells. To further explore the role of PRDX6-iPLA2 in the regulation of mitochondrial fission, fusion-related factors and fission-related proteins were evaluated. In co-culture CTX-TNA2 cells, OGD/R elevated the expression of Mff and Fis1; contrastingly, PRDX6^D140A^ CTX-TNA2 cells expressed less Mff and Fis1, the expression of Mff and FIS1 was further decreased under treatment with NCA. Compared to co-culture CTX-TNA2 cells following OGD/R, PRDX6^D140A^ CTX-TNA2 cells contained more mitochondrial fusion elements Mfn1 and Opa1. The expression of Mfn1 and Opa1 further increased after treatment with NCA (Fig. 6d). These results illustrate that PRDX6-iPLA2 promotes Drp1-dependent mitochondrial fission increasing ROS accumulation following OGD/R.

Interestingly, Drp1-induced excess mitochondrial fission also increases the generation of excess ROS [2]. To determine whether mitochondrial fission in CTX-TNA2 cells also resulted in the accumulation of ROS, a ROS activity test was performed to detect the concentration of ROS in CTX-TNA2 cells and their supernatants after treatment with the mitochondrial fission inhibitor Mdivi-1 (Fig. 6e). Compared to that in PRDX6D140A CTX-TNA2 cells following OGD/R stimulation, ROS generation was further reduced after treatment with Mdivi-1. This result suggests crosstalk between Drp1-dependent mitochondrial fission and the generation of ROS regulated by PRDX6-iPLA2 following OGD/R stimulation.

### Phosphorylation of astrocytic PRDX6 mediates NOX2 activation by regulating its translocation and gaining iPLA2 activity in astrocytes after OGD/R

**Fig. 7:**
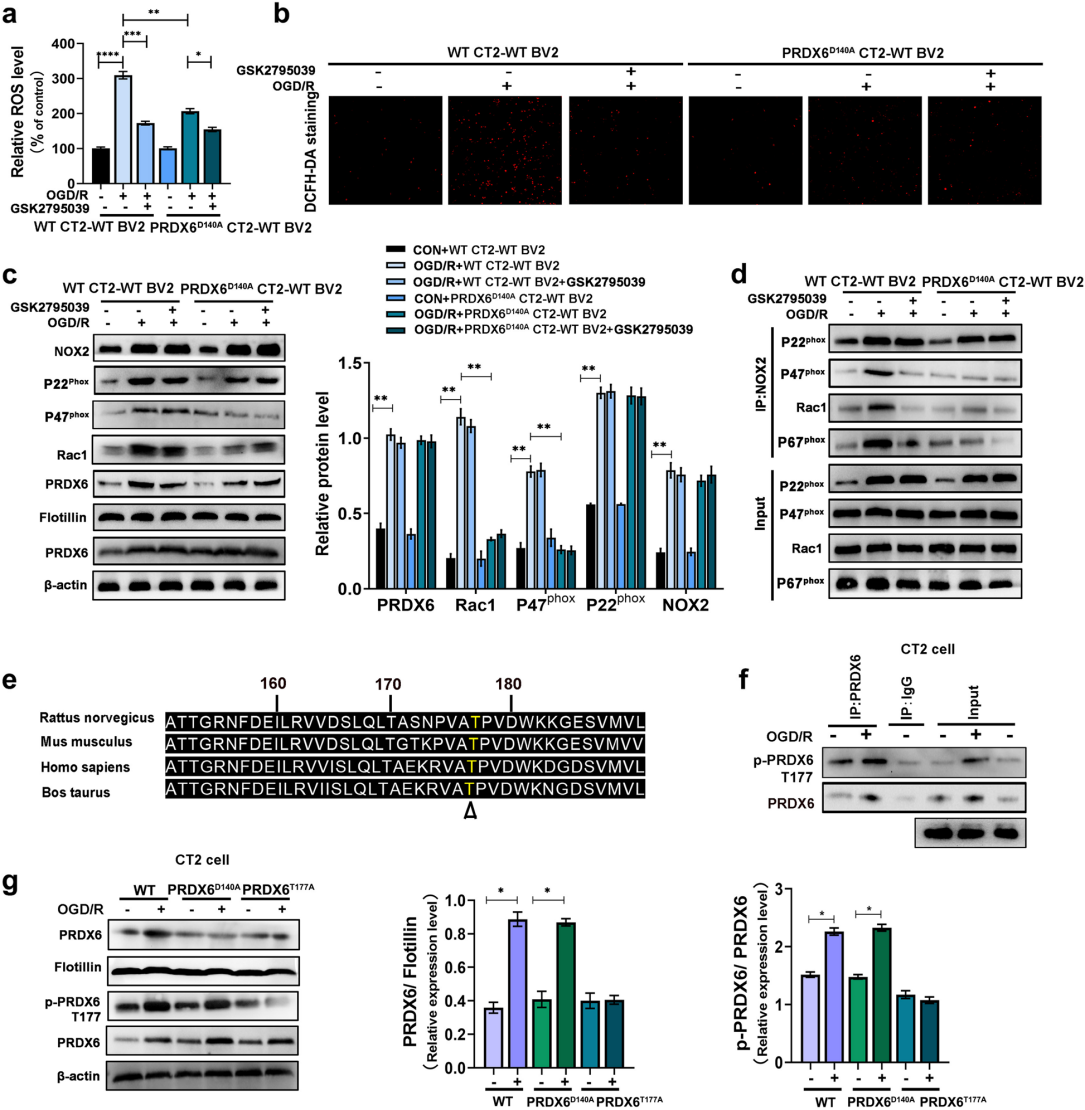
Phosphorylation of PRDX6 mediates NOX2 activation by regulating its translocation and gaining iPLA2 activity in astrocytes.** a** Quantification of ROS. **b** DCFH-DA staninig. **c** Western blot analysis of membranous PRDX6, NOX2, P22^phox^, P47^phox^, Rac1, and total PRDX6 levels. **d** Immunoprecipitation and immunoblot analyses of the interaction between P22^phox^, P47^phox^, Rac1 and P67^phox^. **e** PRDX6 peptides. **f** Immunoprecipitation and immunoblotting analyses of PRDX6 phosphorylation. **g** Western blot analysis of membranous PRDX6, total PRDX6, and p-PRDX6 levels. Values are mean ± SEM, *p < 0.05, **p < 0.01, ***p < 0.001, ****p < 0.0001

NADPH oxidase 2 (Nox2), a part of the NADPH oxidase complex, is a major source of reactive oxygen species (ROS) [43]. Thus, we examined whether PRDX6-iPLA2 interaction regulates ROS production through NOX2 activation in CTX-TNA2 cells following OGR/R stimulation. Compared to PRDX6^D140A^ CTX-TNA2 cells following OGD/R stimulation, ROS generation was further reduced after treatment with the NOX2 inhibitor GSK2795039 (Fig. 7a, b).

Upon activation of NOX2, p47^phox^ is translocated from the cytoplasm to p22^phox^ in the cell membrane, recruiting the additional components p67^phox^, and Rac GTPases, to assemble a complex with NOX2 [20]. We verified that there was increased translocation of Rac1, p47^phox^, and p67^phox^ with an upregulation of p22^phox^ following OGD/R in co-culture CTX-TNA2 cells. The NOX2 inhibitor GSK2795039 had no effect on the translocation of Rac1, p47^phox^, or p67^phox^. Interestingly, the translocation of Rac1, p47^phox^, and p67^phox^ was markedly inhibited in PRDX6^D140A^ CTX-TNA2 cells, but this change was not affected by the NOX2 inhibitor, GSK2795039. However, changes in PRDX6 and NOX2 levels had no effect on the levels of p22^phox^ (Fig. 7c). Next, immunoprecipitation of the membranes isolated from cells under basal conditions showed weak interactions among Rac1, p47^phox^, p67^phox^, and p22^phox^ in CTX-TNA2 cells. OGD/R promoted this interaction, which was reversed by treatment with the NOX2 inhibitor, GSK2795039 (Fig. 7d). Compared with OGD/R + WT CT2-WT BV2 group, no significant association of these proteins was observed in PRDX6^D140A^ CTX-TNA2 cells, consistent with treatment with the NOX2 inhibitor GSK2795039 under these conditions. These data suggest that iPLA2 activity of PRDX6 plays an important role in NOX2 activation in astrocytes.

**Fig. 8:**
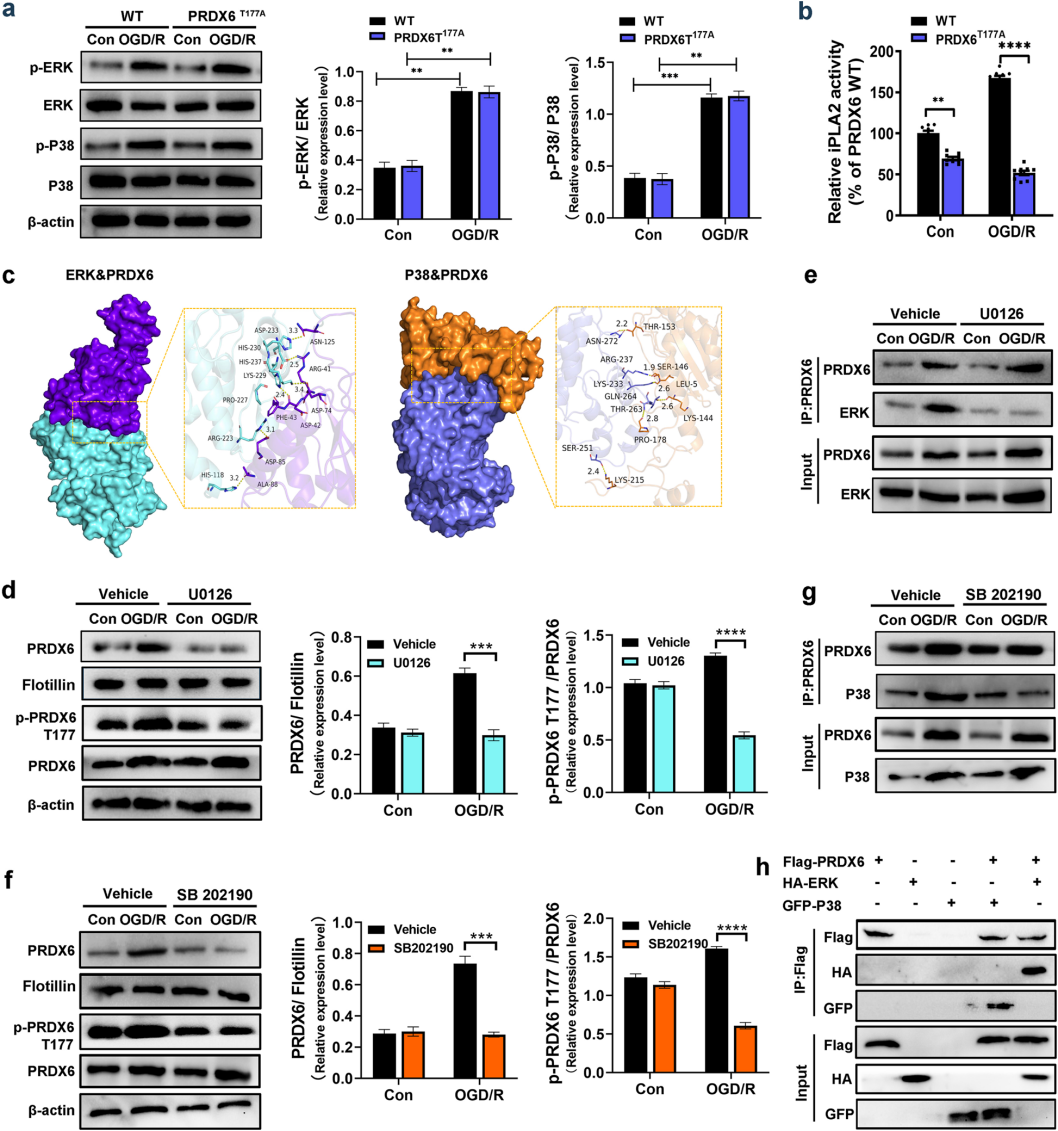
MAPK activity mediates phosphorylation of PRDX6 and its translocation to the cell membrane in astrocytes after OGD/R.** a** Western blot analysis of ERK, p-ERK, P38 and p-P38 in CTX-TNA2 cells. **b** iPLA2 activity in CTX-TNA2 cells. **C** Molecular docking of ERK with PRDX6, P38 with PRDX6. **d**,** f** Western blot analysis of PRDX6, p-PRDX6, membranous PRDX6. **e** Immunoprecipitation and immunoblot analyses of ERK-PRDX6 in CTX-TNA2 cells. **g** Immunoprecipitation and immunoblot analyses of P38-PRDX6 in CTX-TNA2 cells. **h** Immunoprecipitation and immunoblot analyses of ERK-PRDX6 or P38-PRDX6 in HEK293T cells. Values are mean ± SEM, *p < 0.05, **p < 0.01, ***p < 0.001, ****p < 0.0001. *n* = 6 per group

We explored whether NOX2 activation is mainly regulated by the translocation of PRDX6 from the cytoplasm to the plasma membrane by measuring total PRDX6 expression in the cell plasma membrane. Western blotting revealed that PRDX6 was translocated to the cell plasma membrane in co-culture CTX-TNA2 cells following OGD/R. GSK2795039, a NOX2 inhibitor, did not affect the translocation of PRDX6 (Fig. 7c). Likewise, downregulation of PRDX6-iPLA2 activity by the D140A mutant had no effect on PRDX6 translocation induced by OGD/R (Fig. 7c). These results indicate that PRDX6 translocation is independent of PRDX6-iPLA2 activity and may be an upstream mechanism involved in the regulation of PRDX6-iPLA2 activity. A previous study showed that phosphorylation of PRDX6 at Thr177 is required for the translocation of PRDX6 to the plasma membrane, and that P-PRDX6 obtains the activity of LPA2 [13]. The Thr177 site of PRDX6 is evolutionarily conserved in PRDX6 proteins of various species, including mice, humans, and rats (Fig. 7e). To confirm whether Thr177 of PRDX6 was phosphorylated after cerebral ischemia, we immunoprecipitated PRDX6 from cells after OGD/R and probed the immunocomplexes using antiphosphorylation. As shown in Fig. 7f, anti-phosphorylation specifically recognized immunoprecipitated PRDX6 from OGD/R-stimulated CTX-TNA2 cells, but not from control CTX-TNA2 cells. Phosphorylated PRDX6 antibody was used to evaluate PRDX6 phosphorylation in astrocytes. The phosphorylation of PRDX6 was significantly upregulated in wild-type CTX-TNA2 cells after OGD/R. Downregulation of PRDX6-iPLA2 activity in the D140A mutant had no effect on the phosphorylation of PRDX6 following OGD/R. Weak PRDX6 phosphorylation was seen in PRDX6^T177A^ CTX-TNA2 cells under OGD/R stimulation (Fig. 7g). Western blotting also showed reduced translocation of PRDX6 from the cytoplasm to the plasma membrane in PRDX6^T177A^ CTX-TNA2 cells after OGD/R treatment (Fig. 7g). The PRDX6-iPLA2 activity was reduced in PRDX6^T177A^ CTX-TNA2 cells (Fig. 8b). Thus, the phosphorylation of PRDX6 at Thr177 is required for the translocation of PRDX6 and its iPLA2 activity.

### MAPK activity mediates phosphorylation of PRDX6 and its translocation to the cell membrane in astrocytes after OGD/R

The phosphorylation of cytosolic PRDX6 is mainly regulated by MAPK (ERK or p38) activity [44]. MAPK (ERK or p38) and PRDX6 peptides were designed with HDOCK software using structures/sequences of MAPK (ERK or p38) and PRDX6 obtained from the Uniport Database. ERK and P38 interacted with PRDX6 by interacting with multiple residues (Fig. 8c). We determined whether MAPK (ERK or p38) was involved in the phosphorylation of cytosolic PRDX6 and its iPLA2 activity. We first detected ERK and P38 phosphorylation in wild-type cells under OGD/R stimulation. We found that the phosphorylation of ERK and P38 was significantly upregulated in wild-type cells following OGD/R. Downregulated phosphorylation of PRDX6 in the T177A mutant did not affect the phosphorylation of ERK and P38 induced by OGD/R (Fig. 8a). Next, U0126 and SB 202190 were used to inhibit the activation of ERK and p38, respectively. We found that treatment with U0126 or SB 202190 significantly downregulated the phosphorylation of PRDX6 and translocation of PRDX6 after OGD/R in wild-type cells (Fig. 8d, f). Immunoprecipitation results showed that ERK or p38 had a stronger interaction with PRDX6 in wild-type cells after OGD/R whereas, after treatment with U0126 or SB 202190, no significant interaction of these proteins was observed in wild-type cells (Fig. 8e, g). To further identify the interactions among these proteins, we transfected HEK293T cells with the target overexpression plasmids and used them for co-immunoprecipitation (Co-IP) experiments. As shown in Fig. 8H PRDX6 is associated with ERK and p38.

## Discussion

Microglia and astrocytes are key regulators of neuroinflammation and respond as one unit when the brain is perturbed [39]. Interestingly, in addition to participating in neuroinflammation, astrocytes are one of the most prominent contributors to balance oxidative stress, making this response unique [34]. Although astrocytes are rich in GSH and GSH metabolism-related enzymes [14, 21], they release large amounts of ROS to balance oxidative stress. ROS-induced signaling plays a role in the onset and amplification of inflammation [48]. Once the dynamic balance in the regulation of oxidative stress in astrocytes is disrupted, neuroinflammation may be aggravated in CNS diseases [52]. A recent study showed that astrocyte-derived IL-33 is involved in regulating the pruning of microglia-associated synapses and increasing the expression of chemokines [40]. Several studies have revealed that activated astrocytes can regulate reactive microglia to transform into the M1 phenotype via various cytokines [23]. ROS play an important role in activating microglia/infiltrating macrophages and mediating their phenotype. Thus, astrocytes release large amounts of ROS in CNS diseases. We speculated that astrocytes induce M1 microglial alterations by generating and releasing ROS. Our study revealed, in co-culture system treatment with NAC after OGD/R could reduce the number of M1 microglia. In light of these previous studies, the results of our study further supplement the regulatory mechanisms of astrocytes in microglia and neuroinflammation. Another study has shown that in the central nervous system, lipopolysaccharide- stimulated secretion of IL-1α, C1q, and TNFα in microglia induce A1 reactive astrocytes [22]. Thus, there appears to be a strong relationship between microglia and astrocytes and microglia-astrocyte crosstalk plays an important role in regulating the progression of cerebral I/R injury.

PRDX6, a “moonlight protein,” plays the roles of both glutathione peroxidase and calcium-independent phospholipase A2 (iPLA2). GSH peroxidase activity plays an important role in protecting against oxidative stress, while iPLA2 activity mainly participates in the generation of ROS [11]. And astrocytes also play a dual role in the regulation of oxidative stress and neuroinflammation. Therefore, we speculated that PRDX6 is a key molecule in astrocytic functions that regulate the crosstalk between neuroinflammation and oxidative stress. Additionally, astrocyte-derived ROS can amplify neuroinflammation [30]. Although PRDX6 has dual functions, it is reasonable to assume that the activity of the PRDX6-iPLA2 complex regulates neuroinflammation in vivo. Accumulating evidence has demonstrated that PRDX6 is mainly expressed in astrocytes [3]. In our study, we found that PRDX6 protein and PRDX6-iPLA2 activity were remarkably upregulated in astrocyte in cerebral I/R injury. Increased ROS production and upregulation of PRDX6 were observed after cerebral ischemia injury, implying that oxidative stress induced by cerebral ischemia leads to increased transcription of PRDX6. Subsequently, upregulation of PRDX6-iPLA2 activity increases the generation of ROS. Similar to previous studies [29, 32], immunofluorescence staining showed that PRDX6 was expressed in astrocytes, but not microglia, in the ischemic penumbra following MCAO. However, western bolt showed that PRDX6 is mainly expressed in astrocytes, but less expression in microglia. And the changes in expression and iPLA2 activity of PRDX6 in astrocytes more significant than in microglia. The reason may either be that the low content of PRDX6 protein could not be detected by immunofluorescence in microglia or that transcription of PRDX6 was inhibited in microglia. However, another study showed that PRDX6 debris co-localized with the cell membranes of F4/80 + macrophages, which may be related to PRDX6 release from dying cells and subsequent engulfment by macrophages [38]. Whether or not PRDX6 exists in microglia, it does not show any obvious changes after cerebral ischemia. We speculated that this part of PRDX6 is not the main factor regulating microglial polarization. Thus, we speculate that the regulation of PRDX6-iPLA2 on microglia polarization may be related to the activation of astrocytes. Next, we compared the effects of the inhibition of PRDX6-PLA2 in different cells on microglia polarization in the monculture system and the co-culture system, and we found that the inhibition of PRDX6-iPLA2 in astrocytes had the greatest effect on the microglia polarization. Here, we also provide novel evidence that MJ33, a PRDX6-iPLA2 inhibitor, relieves middle cerebral artery occlusion (MCAO)-induced pathological brain injury, regulates astrocyte activation, and reverses microglia/infiltrated macrophage polarization in vivo. In vitro, PRDX6 ^D140A^ cell showed low ROS concentrations after OGD/R and OGD/R-stimulated PRDX6^D140A^ astrocyte supernatant inhibited M1 microglia/infiltrated macrophage polarization. Thus, PRDX6-iPLA2 in astrocytes plays a regulatory role in microglia/infiltrated macrophage polarization by generating and releasing ROS.

In the normal state, mitochondrial dynamics maintain a balance between ROS production and elimination. Mitochondrial dynamics involve repeated fusion and fission, with mitochondrial fission leading to mitochondrial fragmentation and increased ROS generation [2]. A previous study showed that the balance of mitochondrial division/fusion in astrocytes increases ROS formation [26]. In the current study, the mitochondrial fission inhibitor Mdivi-1 remarkably inhibited ROS generation in astrocytes. Conversely, excessive ROS can induce mitochondrial fission in activated glia, impair glial function, and enhance neuroinflammatory responses via various signaling pathways in CNS diseases [8]. Previous studies have shown that ROS are the upstream regulators of DRP1 phosphorylation. Mitochondrial fission is mainly regulated and controlled by Drp1, located mainly in the cytoplasm and translocated to the mitochondria [15], fission protein 1 (Fis1), and mitochondrial fission factor (Mff) on the outer mitochondrial membrane function as a mitochondrial adaptor protein for DRP1 [6, 8, 33]. Additionally, the translocation of DRP1 to the mitochondria and activation of fission are driven by phosphorylation at Ser616 in the GTPase effector domain. In the current study, we found that phosphorylation of Drp1 at Ser616 and the expression of mitochondrial Drp1, Fis1, and Mff were reduced, and the expression of cytoplasmic Drp1 was increased in astrocytes treated with NAC following OGD/R. Thus, there appears to be strong crosstalk between mitochondrial dynamics and ROS production in astrocytes.

Accumulating evidence has shown a pivotal role for PRDX6 in mitochondrial homeostasis [24], and our previous study also found that the suppression of PRDX6 could enhance mitophagy and apoptosis following cerebral I/R injury. Mitochondrial fragmentation occurs before the final step of apoptosis [16, 19], suggesting that PRDX6 regulates the dynamic mitochondrial network. In the current study, we found that the expression of PRDX6 and mitochondrial fission-related factors Drp1, Fis1, and Mff showed the same upregulation trend, whereas the expression of mitochondrial fusion-related proteins showed a negative correlation. Interestingly, another study showed that the suppression of PRDX6 promotes ubiquitin-proteasome (UP)-dependent degradation of Mfn1 and alters the mitochondrial dynamic network [28]. Therefore, we speculate that the role of PRDX6 in promoting mitochondrial fission after cerebral ischemia is related to the activity of iPLA2 and ROS generation. Here, the inhibition of PRDX6-iPLA2 reduced the phosphorylation of Drp1 at Ser616 and the expression of mitochondrial Drp1, Fis1, and Mff, thereby increasing the expression of mitochondrial Mfn1 and OPA1 in astrocytes. The changes in these proteins were further increased after treatment with the ROS inhibitor NCA. Thus, PRDX6 iPLA2 activity in astrocytes plays a vital role in ROS-induced mitochondrial fission induced by ROS after cerebral ischemia.

The NADPH oxidase (NOX) complex is primarily responsible for the generation of ROS associated with inflammation and NOX2 especially plays a vital role in innate immunity [43]. Although NOX2 expression is widely distributed, NOX2 is primarily responsible for ROS generation associated with inflammation in astrocytes [46]. The NOX2 complex consists of intrinsic membrane subunits (gp91phox/Nox2 and p22phox) and cytosolic subunits (p40^phox^, p47^phox^, p67^phox^, and the small GTPase Rac1), which are restricted to their respective compartments [30]. Activation of the enzyme complex requires the translocation of the cytosolic component to the plasma membrane and subsequent association with gp91^phox^/Nox2 and p22^phox^, leading to superoxide generation. In most studies, ROS generation in endothelial cells is mainly regulated by NOX2. Furthermore, the activity–PRDX6-iPLA2 is a key molecule in NOX2 activation [7]. PRDX6–iPLA2 regulates the assembly of NOX2 components on the cell membrane by generating lysophosphatidylcholine (LPC), catalyzing LPC to lysophosphatidic acid (LPA), and binding it to its receptor (LPAR). This results in Rac phosphorylation, which in turn enables the assembly of the cytosolic components with the membrane components. However, it is unclear if the activity of PRDX6-IPLA 2 results in NOX2 activation in other cells. Here, we generated PRDX6^D140A^ astrocytes to establish a cell model of cerebral I/R injury, we verified that translocation of Rac1, p47phox, and p67phox was markedly inhibited and that interaction of these subunits in the PRDX6^D140A^ cells was weak, a change further enhanced by the NOX2 inhibitor GSK2795039. Thus, PRDX6-iPLA2 activity is independent and presumably upstream of the NOX2 activation pathway. Combined with previous studies on regulating NOX2 on Drp1-dependent mitochondrial fission, we speculated that PRDX6-iPLA2 activity affects mitochondrial fissions via NOX2.

Previous studies have shown that mitogen-activated protein kinases regulate PRDX6-iPLA2 activity by phosphorylating PRDX6 at Thr177 sites [44]. MAPKs preferentially catalyze the phosphorylation of the Ser/Tr-Pro sequence, which is easily recognized by the active site of the kinase [4]. Here, we showed that the Thr177 sequence of PRDX6 is conserved in different species. ERK and P38 activities increased in astrocytes after OGD/R. However, the activities of ERK and P38 induced by OGD/R did not change in Thr177 mutant astrocytes. Additionally, both ERK and P38 affect the phosphorylation of PRDX6 at Thr177 in astrocytes, thereby increasing iPLA2 activity.

## Conclusion

**Fig. 9:**
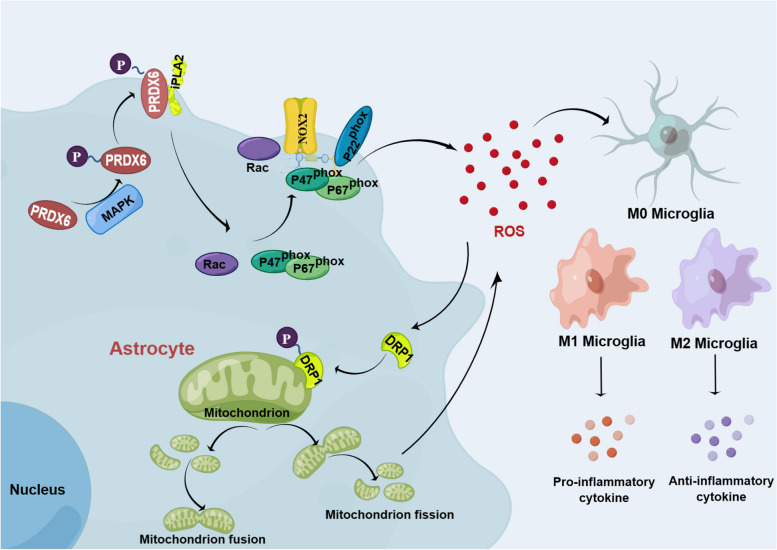
Mechanisms of a novel role for PRDX6 and iPLA2 in regulating astrocyte-induced ROS production and ROS-induced microglia/infiltrated macrophage polarization by activating the NOX2 and Drp1‑mitochondrial fission pathways. Further, PRDX6-iPLA2 activity is regulated by ERK and P38 via phosphorylation of PRDX6 at Thr177 in astrocytes

In summary, as shown in the Fig. 9, this study identified a novel role for PRDX6 and iPLA2 in regulating astrocyte-induced ROS production and ROS-induced microglia/infiltrated macrophage polarization by activating the NOX2 and Drp1‑mitochondrial fission pathways. Further, PRDX6-iPLA2 activity is regulated by ERK and P38 via phosphorylation of PRDX6 at Thr177 in astrocytes.

## Data Availability

All data supporting the conclusions of this article are included within the article.
